# US–China cooperation and competition in science and technology

**DOI:** 10.1093/nsr/nwaf001

**Published:** 2025-01-20

**Authors:** Mu-ming Poo

**Affiliations:** Scientific Director, Institute of Neuroscience, Chinese Academy of Sciences, China

On 13 December 2024, the US and Chinese governments amended the Science and Technology Agreement (STA) and extended it for five years. This 45-year-old agreement was one of the first inter-governmental agreements following the establishment of US–China diplomatic relations in 1979. Since then, the STA has been renewed every five years and has enabled collaborative projects between governmental agencies in agriculture, renewable energy, climate science, health science and biotechnology. This STA extension should be beneficial to both countries and international communities, but its significance and impact remain uncertain, given the growing geopolitical and economic tension and the lack of basic trust between the two governments.

The renewed STA addresses cooperation in non-sensitive areas in environmental science, public health and agriculture, but excludes emergent technologies such as quantum computing, artificial intelligence and cybersecurity, due to national security concerns. The critical issue is the balance between cooperation and competition. Many global problems faced by humanity, from pandemics to global warming, require cooperative efforts among all societies, especially the two largest economies, which play leading roles in science and technology (S&T). While the exclusion of some emerging technologies is understandable, the definition of the areas that carry a national security risk is often vague, arbitrary and subject to change. The cooperative spirit is also undermined by the assumption that winning the competition is the only goal.

As a researcher actively working in several competitive fields over the past decades, I have benefited greatly from both collaboration and competition with many colleagues. Through collaboration, we were able to solve difficult problems that were not attainable by my own group alone. On the other hand, competition with other groups mobilized the spirit of innovation and elevated the aspiration for higher achievement. Notably, competitive peers often attained similar goals via different routes of experimentation and innovation, and results were often published as back-to-back papers in scientific journals. Both were winners of the competition, and the field benefited as a whole.

The renewed STA provides new guardrails for protecting the safety and security of researchers. This represents an important aspect of the STA. For example, a large number of US scientists of Chinese origin have been a driving force in the past for US–China collaborations, which benefited S&T development in both countries. Yet, since the instigation of the ‘China Initiative’ by the US government, there have been more than 200 cases of harassment, un-justified investigations and ill-treatment of scientists of Chinese origin. Restrictions on travel and scientific communications for US scientists with their Chinese colleagues remain in place, having a chilling effect on scientific collaboration. Creating an encouraging atmosphere and protective mechanism for scientists willing to collaborate is a prerequisite for the STA to achieve its goal.

The STA also stipulates new provisions for ensuring transparency and reciprocity in data sharing. Data sharing is the basis for all successful collaborations, and mechanisms have been worked out for many existing international projects in medicine, physics and astronomy, in which Chinese scientists have participated. A critical issue is equity in data sharing and credit allocation. I personally know several Chinese scientists who are now reluctant to share their data with US colleagues because their contributions were poorly recognized in previous collaborations by their collaborators in joint publications and in news releases. Equity-based collaboration is incompatible with the notion that US scientists should always lead the collaboration and provide the ideas while Chinese scientists serve to collect the data and make the data accessible for sharing.

The STA is a diplomatic gesture designed to promote cooperation between the two countries, but its focus on intellectual property (IP) protection could be viewed with skepticism. By picturing China as the primary offender in IP disputes, the US not only deflects attention from its own role in perpetuating inequitable IP systems but also uses the IP issue as a tool for geopolitical leverage. Under the framework of the STA, US and China should work together to reform the global IP system and ensure that it fosters innovation, equity and access for all nations.

The extension of the STA at this time signifies the willingness of US and Chinese governments to continue the dialogue on S&T issues. The STA contributed significantly in the past by providing a framework for fruitful collaborations among institutions and laboratories from the two countries. Maintaining these activities is desirable for both countries, at least in basic areas without national security concerns. Although the extended STA is mainly concerned with cooperation between corresponding governmental agencies, it will impact future collaborations at the level of academic institutions and individual scientists. This is clearly a positive move by the two governments at this uncertain time.

## Notes

Executive Editor-in-Chief of NSR

